# Enzymatic Hydrolysis Modifies Emulsifying Properties of Okra Pectin

**DOI:** 10.3390/foods11101497

**Published:** 2022-05-20

**Authors:** Ibukunoluwa Fola Olawuyi, Jong Jin Park, Gwang Deok Park, Won Young Lee

**Affiliations:** 1 School of Food Science and Biotechnology, Kyungpook National University, Daegu 41566, Korea; ifolawuyi@knu.ac.kr (I.F.O.); bjj1490@nate.com (J.J.P.); kbg01015@naver.com (G.D.P.); 2 Coastal Agricultural Research Institute, Kyungpook National University, Daegu 41566, Korea; 3 Research Institute of Tailored Food Technology, Kyungpook National University, Daegu 41566, Korea

**Keywords:** okra pectin, enzyme modification, molecular structure, emulsifying properties

## Abstract

Okra pectins (OKPs) with diverse structures obtained by different extraction protocols have been used to study the relationship between their molecular structure and emulsifying properties. A targeted modification of molecular structure offers a more rigorous method for investigating the emulsifying properties of pectins. In this study, three glycoside hydrolases, polygalacturonase (PG), galactanase (GL), and arabinanase (AR), and their combinations, were used to modify the backbone and side-chains of OKP, and the relationships between the pectin structure and emulsion characteristics were examined by multivariate analysis. Enzymatic treatment significantly changed the molecular structure of OKP, as indicated by monosaccharide composition, molecular weight, and structure analysis. GL- and AR- treatments reduced side-chains, while PG-treatment increased side-chain compositions in pectin structure. We compared the performance of hydrolyzed pectins in stabilizing emulsions containing 50% *v*/*v* oil-phase and 0.25% *w*/*v* pectin. While the emulsions were stabilized by PG (93.3% stability), the emulsion stability was reduced in GL (62.5%), PG+GL+AR (37.0%), and GL+AR (34.0%) after 15-day storage. Furthermore, microscopic observation of the droplets revealed that emulsion destabilization was caused by flocculation and coalescence. Principal component analysis confirmed that neutral sugar side-chains are key for long-term emulsion stabilization and that their structure explains the emulsifying properties of OKP. Our data provide structure-function information applicable to the tailored extraction of OKP with good emulsification performance, which can be used as a natural emulsifier.

## 1. Introduction

Pectins from various sources are promising natural additives in the food, pharmaceutical, and cosmetic industries because of their good gelling, stabilizing, and emulsifying properties [1]. Recently, the direct application of pectins as natural food ingredients has substantially increased the need for further investigation of their distinct functionalities as alternative food emulsifiers [2]. In the food industry, certain products require emulsifiers to ensure the long-term stability of the dispersed oil/water phase during processing and storage, and to enhance the taste and texture of products [3]. Pectins can stabilize oil-in-water (O/W) emulsions because of their surface activity, charge density, and ability to increase the viscosity of the continuous aqueous phase of the emulsion. This reduces interfacial tension, oil droplet aggregation, and mobility [4,5,6]. However, these properties vary depending on the molecular structure of the pectins [1].

Most commercial pectins are extracted from citrus peel and apple pomace, and in a few cases are obtained from sugar beet pulp. Recently, pectin obtained from okra (*Abelmoschus esculentus* L.) pods has gained much interest owing to its unique fine structures, high acetyl content, highly branched structures [7,8,9], and strong biological activities such as its antioxidant [10], anti-hyperglycemic [11], anti-hyperlipidemic, and anti-diabetic effects [12]. Moreover, it has high surface activity and emulsifying performance [8,13,14]. The emulsifying properties of common pectins extracted from apple and citrus are inferior to those of okra pectin (OKP) because of their low protein and acetyl content, high linearity, and absence of extensive branching [2,15], which contribute to the interfacial surface activity and emulsification performance of pectins [1,16,17].

Previous studies of the emulsion activity and stability of OKP indicated the dependence on the molecular structure, which influences diverse functionalities. Moreover, extraction protocols and factors, including extraction pH [18,19], solvent and buffer solutions [7], pre-treatments and drying techniques [9,20], extraction methods [10], and genotype differences [21] impact structural and functional diversity of OKP. The molecular structure of OKP consists of a covalently linked homogalacturonan (HG) region, which is a repeating unit of α-(1,4)-linked galacturonic acid residues (4-α-Gal*p*A-(1,2)-α-l-Rha*p*-1-), and a branched rhamnogalacturonan-I (RG-I) region containing repeating disaccharide α-(1,2)-linked rhamnose and α-(1,4)-linked galacturonic acid residues [11]. The rhamnose in RG-I units is often branched at the *O*-4 position with the neutral sugars galactose and arabinose [7].

The emulsifying properties of OKP have been attributed to structural factors, including its hydrophobic moieties (protein, acetyl, and methyl groups), neutral sugar and backbone (HG: RG-I) composition, and other macromolecular properties including molecular weight and viscosity [2,22]. Previous studies have analyzed the structure–function relationships by correlating emulsifying properties with structural variants of OKP (e.g., linearity, degree of branching) obtained by different extraction protocols [9,14,20,23]. However, a more detailed understanding of these relationships can be provided using the targeted modification of specific molecular structures.

Physical, chemical, and enzymatic treatments can change polysaccharide structures and improve emulsifying properties [24]. Among such methods, enzymatic modification is often preferred because of its specificity and ease of application, and has been used to explore various functionalities of pectins, such as their emulsifying properties [16,25]. Targeted enzymatic modification of pectins from other sources has previously been applied for exploring the influence of specific structures on emulsifying properties [4,16,26,27]. Purified enzymes such as *endo*-polygalacturonase, *endo*-1,4-β-d-galactanase, and *endo*-1,5-α-l-arabinanase have been used to modify structures of sugar beet pectins to provide a better understanding of the contributions of molecular structures on the emulsification performance of pectins [27,28]. In addition, key structural units and mechanisms for emulsification were described. To the best of our knowledge, the impact of enzymatic modification on the emulsifying properties of OKP remains unknown.

To provide a better understanding of the contributions of the molecular structures of OKP to emulsifying stability, three purified glycoside hydrolases, namely polygalacturonase (PG), galactanase (GL), and arabinanase (AR), as well as their combinations, were used to modify the HG backbone and neutral side-chain structures of OKP. Furthermore, multivariate analysis revealed the correlations between structural factors and emulsion characteristics.

## 2. Materials and Methods

### 2.1. Materials

Fresh green okra pods were purchased from a local farm (Dangjin-si, Chungcheongnam-do, Korea). The cleaned pods were sliced horizontally and dried at 40 °C for 48 h. After drying, the pods were pulverized into powder using a laboratory grinder (RT-04, Mill Powder Tech., Tainan, Taiwan), sieved (425 μm aperture; Chung Gye Sang Gong Sa, Seoul, Korea) and stored at –22 °C until extraction. Purified enzymes, including *endo*-polygalacturonase (EC 3.2.1.15, 1100 U/mL, from *Aspergillus aculeatus*), *endo*-1,4-β-d-galactanase (EC 3.2.1.89, 1300 U/mL, from *Aspergillus niger*), β-galactosidase (EC 3.2.1.23, 4000 U/mL, from *Aspergillus niger*), *endo*-1,5-α-l-arabinanase (EC 3.2.1.99, 250 U/mL, from *Aspergillus niger*), and α-l-arabinofuranosidase (EC 3.2.1.55, 300 U/mL, from *Aspergillus niger*) were purchased from Megazyme Ltd. (Wicklow, Ireland). Other chemicals, including sodium acetate, acetic acid, trifluoroacetic acid (TFA), 1-phenyl-3-methyl-5-pyrazolone (PMP), monosaccharide standards, and other analytical chemicals, were purchased from Sigma Aldrich (St Louis, MO, USA).

### 2.2. Extraction of Okra Pectin

Before pectin extraction, okra powder was washed twice with 85% aqueous ethanol solution (1:10 *w*/*v*) at 40 °C, filtered, and the residue was air-dried to obtain an alcohol-insoluble residue (AIR). After that, AIR was extracted twice (1:50 *w*/*v*) using a hot acetate buffer solution (0.05 mM, pH 4.5) at 80 °C for 1 h. The supernatant containing pectin was separated by high-speed centrifugation (7000× *g*, 30 min) and filtered through a cloth until a clear solution was obtained. Pectin was precipitated using 95% ethanol (1:3 *v*/*v* at 4 °C for 12 h), and dried using a freeze-drier (FDS8518, Ilsin BioBase Co. Ltd., Dongducheon-si, Korea) with working conditions of temperature and vacuum pressure of −55 °C and 21–25 Pa, respectively, for 72 h. Dried gum was ground to obtain okra pectin powder (OKP) [9,13]. The sugar composition of OKP was determined as 92.06 ± 1.75% using the phenol-sulphuric method [29].

### 2.3. Enzymatic Modification of OKP

The enzymatic treatments used in this study are listed in Table 1. The glycoside hydrolases and *endo*-polygalacturonase were applied to cleave the polygalacturonan backbone, *endo*-galactanase and β-galactosidase as well as *endo*-1,5-α-l-arabinanase and α-l-arabinofuranosidase were applied to cleave the side-chains of galactan and arabinan, respectively [27,30]. In enzymatic modification experiments, OKP (5 mg/mL) was dissolved completely in acetate buffer (0.05 mM, pH 4.5) at 25 °C by mechanical stirring. Enzymes were added as specified in Table 1 and incubated at 50 °C for 24 h. The sample without enzyme addition represents untreated OKP. After treatment, the enzymes were inactivated (90 °C for 5 min) and the hydrolysates were dialyzed (~13 kDa) against distilled water. After dialysis, modified pectin was precipitated using 95% ethanol (1:2 *v*/*v*), washed with 85% ethanol, and lyophilized for 72 h. Dried pectin was crushed in a pestle and mortar, and passed through a 150 μm aperture sieve to obtain a fine powder. The process of enzymatic modification was carried out thrice and the recovery was calculated as the mean percentage ratio of modified pectin powder (g) to OKP used.

### 2.4. Physicochemical and Monosaccharide Analyses

#### 2.4.1. Physicochemical Compositions

The chemical composition of pectin samples, including total protein content [31] and uronic acid [32] composition, were quantified by standard colorimetric methods using bovine serum albumin and galacturonic acid standards, respectively. To determine the degree of methylation (DM) and acetylation (DA), the technique described by Voragen et al. [33] was used and expressed as percentage moles of methanol and acetic acid to uronic acid, respectively. Pectin powder (30 mg) was saponified in 1 mL of 0.4 M NaOH/ isopropanol solution (50/50 *v*/*v*) and kept at room temperature for 2 h. The supernatant was filtered and injected into an HPLC system (HPLC 1260 series, Agilent Technologies, Santa Clara, CA, USA) consisting of a refractive index detector, Rezex ROA-Organic acid H+ (8%) column (150 mm × 4.6 mm). Column elution was performed with 5 mM H_2_SO_4_ at 0.6 mL/min and 50 °C. For the zeta potential determination, pectin samples (0.1% *w*/*v*) were measured using a Zetasizer Nano ZS apparatus (Malvern, UK) at room temperature. The refractive index of the dispersion medium (water) was set at 1.330.

#### 2.4.2. Monosaccharide Analysis

The sugar composition of pectin was determined after hydrolysis in 2M TFA (121 °C for 2 h) and PMP derivatization, as described in a previous study [34]. The derivatized sample was washed thrice with chloroform to remove excess reagent, and the supernatant was filtered through a 0.45 μm membrane. The filtrate (20 μL) was injected into the HPLC system (Jasco International Co., Ltd., Tokyo, Japan) equipped with an Athena C18 reverse-phase column (250 mm × 4.6 mm, 5 μm) and detected using a UV/VIS detector (UV-2075 plus, Jasco International Co., Ltd., Tokyo, Japan) at 245 nm. Column elution was performed at a flow rate of 1 mL/min with potassium phosphate buffer (0.05 M, pH 6.9) and acetonitrile in a ratio of 83:17 (*v*/*v*, %).

### 2.5. NMR and FT-IR Structural Characteristics

Nuclear magnetic resonance (NMR) analysis was performed using an NMR spectrometer (500 MHz Bruker Ascend™, Billerica, MA, USA). For this analysis, pectin was dissolved in 0.5 mL D_2_O (99.96%), lyophilized for 2 h, and then re-dissolved in D_2_O. This process was repeated twice to replace exchangeable protons, and a clear pectin solution was used to obtain proton ^1^H NMR data using 512 scans at room temperature. Data processing was performed using Mnova v.14.1.2 software (Mestrelab Research) [13]. FT-IR spectra were recorded in the range of 4000−400 cm^−1^ at 4 cm^−1^ resolution using an FT-IR spectrophotometer (Frontier, PerkinElmer, MA, USA). The samples were pressed with KBr for analysis.

### 2.6. Molecular Weight and Apparent Viscosity Determination

The molecular weights of pectins were determined using a Thermo Dionex HPLC Ultimate3000 RI System (Sunnyvale, California, USA) equipped with Waters Ultrahydrogel 120, 500, and 1000 columns. Pectins (5 mg/mL) dissolved in 0.1% sodium azide were filtered through a 0.45 μm membrane, and 50 μL was injected into the system. Elution was carried out at 40 °C using 0.1 M sodium azide in water at a flow rate of 1 mL/min. Pullulan was used as a quantitative standard, and data were processed using the Chromeleon 6.8 Extention-pak software. Apparent viscosity (η_ap_) of pectin solutions (0.5% *w/v*) was measured at different shear rates (γ, 38.4–768 s^−1^) using a Brookfield viscometer (DV-II + PRO, MA, USA) equipped with a spindle (No. 42). Measurements were performed at 25 °C. To explain the flow characteristics, the data were further fitted to the power-law model (Equation (1))

(1) $$\tau =K\gamma^{n}$$

where τ is shear stress, *K* is the flow consistency index, and *n* is the flow behavior index.

### 2.7. Emulsion Preparation and Characteristics

Aqueous solutions of pectins (0.5% *w*/*v*) were prepared by dissolving pectin powder in 0.1 M phosphate buffer (pH 7.0) containing 0.1% sodium azide. The emulsion was then prepared by mixing pectin solution with soybean oil (1:1) to attain a final pectin concentration of 0.25% *w*/*v*. Coarse emulsions were formed by high-shearing homogenization (PT-1200C, KINEMATICA AG, Malters, Switzerland) at 20,000 rpm for 1 min. Subsequently, the emulsions were ultrasonicated (45 kHz, JAC-5020, KODO, Hwaseong, Korea) at 25 °C for 2 min [35].

The color values (L*- lightness, a*- redness, and b*- yellowness) of the fresh emulsions after preparation were measured using a chroma meter (CR-300, Minolta Co., Osaka, Japan) and are presented as lightness (L*) and chroma (C*) values [36]. The measuring head of the CR-300 uses diffuse illumination/0° viewing angle, equipped with a pulsed xenon arc (PXA) lamp and an 8 mm-diameter measuring area. The apparent viscosity of emulsions was recorded at different shear rates (19.2–576 s^−1^) as described in Section 2.6.

For the storage experiment, freshly prepared emulsions were then transferred into calibrated tubes and stored for 15 days at 25 °C. The emulsifying capacity (EC) during storage was determined by measuring the volume of the emulsified layer (*E_v_*) relative to the total volume (*T_v_*) (Equation (2)). Emulsion stability (ES) was calculated as the ratio of the final emulsion layer (*F_ev_*) after storage to the initial emulsion layer (*I_ev_*) (Equation (3)) [37]. The emulsion activity index (EAI) and emulsion turbidity loss (*k*_1_) after storage period were determined turbidimetrically by using a UV spectrophotometer (Shimadzu Co. UV-2550, Tokyo, Japan) [38]. Emulsions were diluted in 0.1% SDS solution, and their absorbance was measured at 500 nm.

(2) $$\mathrm{EC}\;(\%)=\frac{E_{v}}{T_{v}}\;\times \;100$$

(3) $$\mathrm{ES}\;(\%)=\frac{F_{ev}}{I_{ev}}\;\times \;100$$

(4) $$\mathrm{EAI}\;(m^{2}/g)=\frac{4.606\times A_{500}}{C\times \varphi \times 1000}\times DF$$

(5) $$\mathrm{In}A=\mathrm{In}A_{0}-k_{1}t$$

In Equations (4) and (5), DF is the dilution factor (100), *φ* represents oil fraction (0.5), and c denotes the concentration of pectin (g/mL). In Equation (5), A is the absorbance after time t (15 days), *A*_0_ is the absorbance at time 0, and *k*_1_ is the first-order rate constant representing turbidity loss.

The emulsion droplets were observed after the storage period using a digital optical microscope (Leica, MC120 HD, Wetzlar, Germany) equipped with an HD microscope camera. The micrographs were captured at ×200 magnification.

### 2.8. Statistical Analysis

All experiments were performed in triplicates, and the data are presented in tables and graphs as the mean ± standard deviation. Duncan’s multiple range test (*p* < 0.05) was used to separate the means at the 95% significance level using SPSS v.22 software (SPSS Inc., Chicago, IL, USA). Multivariate analysis (PCA) was carried out using STATISTICA v8.0 software (StatSoft Inc., Tulsa, OK, USA).

## 3. Results and Discussion

### 3.1. Enzymatic Treatment and Composition of Pectins

The five pectins, namely four enzyme-treated and one untreated OKP, were obtained as shown in Table 1. The recovery of OKPs varied according to the enzyme treatment (Table 2) because of the depolymerization of the backbone and the partial degradation of side-chain sugars associated with enzymatic treatments [27]. The protein contents of pectin samples are within the close range of 1.53 to 1.66%, and similar to a previous study [18]. This implies that enzymatic treatment did not greatly alter the protein composition of pectins. Proteins in pectin extracts are often considered impurities or integral parts of the polymer, since the primary walls of plant cells contain both polysaccharides and structural proteins [39].

Enzymatic treatments changed the degree of acetylation (DA) (37.70–47.45%) and methylation (DM) (6.05–14.59%) in pectin samples. Acetyl and methoxyl groups are linked to GalA residues in pectin chains by forming reactive ester bonds with the carboxyl and hydroxyl groups [40]. Furthermore, an unusual substitution of rhamnosyl residues with acetyl groups has been reported for OKPs [7]. Pectins obtained from okra pods have been found to be highly acetylated (>15%) with a low degree of methyl-esterification (<50%) [7,13,21]. The variations in DA and DM observed in this study may either be because of different activities of enzymes inducing methoxyl or acetyl group cleavage, or because of the changes in the molar composition of GalA residues. The presence of methyl and acetyl groups has been reported to positively influence emulsion droplet formation and stability, owing to their hydrophobic properties and surface activities [40]. The zeta potential of the pectins was measured at pH 7.0. The negative zeta-potential values of pectins confirmed their anionic nature and varied significantly according to enzyme treatments (−21.15 to −39.60 mV) (Table 2). Variations in zeta potential after enzyme hydrolysis of polysaccharides have been observed in previous studies [24].

In this study, PG-treated pectin showed a slight increase in the absolute zeta potential compared to CON, which is likely because of a lower DM, resulting in a higher amount of free carboxyl groups in the pectin structure [41]. In contrast, PG+GL+AR− and GL+AR-treated pectins had lower absolute zeta potential values (<30 mV) despite their higher GalA composition and lower DM. The reason for this remains unclear. However, a possible explanation is that other factors could have induced conformational changes concealing and suppressing the carboxyl groups on the surface [41]. The charge density of a pectin solution is related to its emulsifying properties [40]. An emulsion stabilized with a pectin solution with a high zeta potential (negative or positive ≥ 30 mV) ensures electrostatic repulsion among pectin chains, resulting in increased inter- and intra-molecular repulsion between oil droplets enveloped in the continuous phase. Thus, ES can be improved by preventing flocculation and coalescence of the emulsion droplets.

### 3.2. Monosaccharide and Molar Ratios

The monosaccharide composition of enzyme-treated pectins was significantly (*p* < 0.05) affected by enzyme type (Table 2). All samples contained a high proportion of galacturonic acid (GalA, 35.85–47.91 g/100 g), which is typical for pectic polysaccharides [42]. Galactose (Gal, 14.40–25.66 g/100 g), rhamnose (Rha 10.03–13.68), and arabinose (Ara, 0.37–1.84%) were identified as the main neutral sugars, whereas glucose (Glc, 0.37–0.88%) and mannose (only detected in CON) were present as co-extracted cell wall oligomers [42]. However, variations in the content of GalA and other neutral sugars were attributed to the enzymatic treatments. For instance, PG-treated pectins had the lowest GalA content, whereas AR and GL decreased arabinose and galactose contents, respectively. In addition, GL- and GL+AR-treated pectins had the highest molar percentage (mol %) of GalA because of a decreased neutral sugar content caused by enzymatic degradation.

The molar ratios (MR_1_–MR_5_) were estimated by the mol% composition of each sugar residue (Table 2, parentheses), providing essential information on the molecular structure of the pectins, as shown in Table 3 [42,43]. MR_1_ represents the contribution of rhamnogalacturonan-I (RG-I) to pectin structure, and all pectins in this study were categorized as RG-I type (Rha/GalA = 0.05–1.0) [30]. However, a high value of 0.38 for PG and 0.34 for PG+GL+AR pectins suggested the existence of a higher RG-I unit in their backbone structure compared to that in CON (0.28) and other enzyme-treated pectins (0.27). Moreover, this result is coherent with their RG-I values (55.37–69.52). In addition, the molar ratio of HG/RG-I indicated that all pectins were complex mixtures of linear homogalacturonan (HG) and branched RG-I, with the latter being the predominant structural unit in all pectins (HG/RG-I < 1). However, hydrolysis of side-chain sugars (Gal and Ara) in GL- (0.76) and GL+AR- (0.80) treated pectins remarkably reduced the RG-I proportion in the pectin structure (Table 3).

Compared to untreated pectin (CON), linearity (MR_5_) was reduced in PG-treated pectin because of the partial cleavage of GalA linkages present in the HG backbone by the endo-PG, whereas enzymatic hydrolysis of Ara and Gal side-chain sugars increased the linearity of GL- and GL+AR-treated pectins. MR_2_ (0.30–0.50), MR_3_ (0.29–0.46), and MR_4_ (0.01–0.04) represented the contribution of total neutral sugars, galactose, and arabinose side-chain sugars to the pectin structure, respectively. We analyzed these molar ratios because of their strong correlation with the emulsifying performance of pectin solutions [14,43]. Thus, the observed variations in these ratios among enzyme-treated pectins can underlie the notable differences in their emulsification performances. Alba et al. [14] compared the emulsifying properties of two OKPs obtained using hot buffer extraction (pH 2 and 6). The authors assessed ES via emulsion interfacial composition and concluded that the pectin fraction with higher neutral sugar side-chain branches formed thicker and more compact interfaces, resulting in greater ES. Furthermore, another study correlated the structural features of 43 sugar beet pectins extracted under varying conditions with their emulsifying properties and concluded that the composition of neutral sugar side-chains of pectins (MR_2_–MR_4_), especially arabinose content, is the principal factor for emulsion stabilization [43].

### 3.3. FT-IR and NMR Characteristics

**Figure 1:**
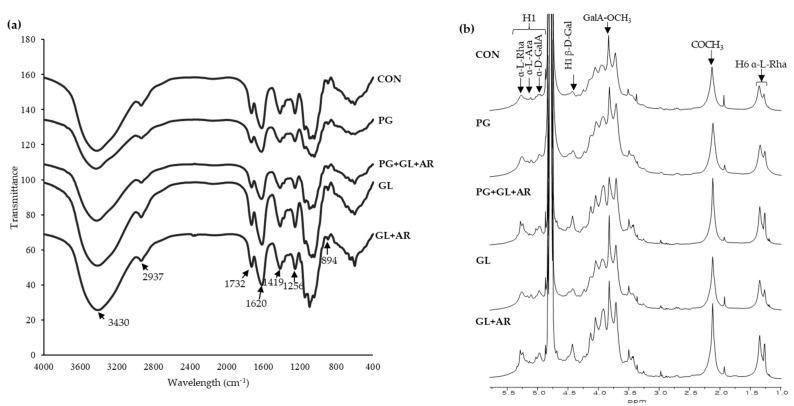
FT-IR (**a**), and ^1^H NMR (**b**) spectra of OKP following enzymatic treatments.

The pectin structures were compared by measuring the characteristic peaks of the functional groups in pectin using FT-IR and NMR spectroscopy. In the FT-IR spectra (Figure 1a), the broad and small peaks at 3430 cm^−1^ and 2937 cm^−1^ correspond to O–H stretching in sugars and C–H stretching in CH_2_ groups, respectively [40]. Furthermore, the characteristic peaks of the carbonyl (C=O) group of the esterified and non-esterified carboxylic groups (–COOH) of uronic acids were detected at 1732 cm^−1^ and 1620 cm^−1^, respectively [44]. These peaks have been previously used for the rapid estimation of the degree of esterification of pectin from various sources [45]. The peak area at 1732 cm^−1^ was smaller than at 1620 cm^−1^, confirming a low degree of esterification in all OKP samples (Table 2), which is consistent with other studies [13,21]. However, slight changes in the peak intensities of enzyme-treated pectins imply that the enzymatic treatment altered the esterification pattern. The peaks at 1419 cm^−1^ and 1256 cm^−1^ were attributed to the symmetric stretching of the carboxylate group (C–OH) and C–O stretching of the acetyl group [30]. The peaks between 1010 and 1150  cm^−1^ (i.e., 1042 , 1092, and 1148 cm^−1^) correspond to the C–O and C–C vibrations of glycosidic bonds and pyranose, and the peak at 894 cm^−1^ suggests the presence of α-type glycosidic linkages [46].

All pectin samples showed similar characteristic peaks of the functional groups, and no new peaks were observed. However, the variations in the peak intensities may be attributed to compositional differences [44].

In ^1^H NMR spectra (Figure 1b), proton peaks were assigned according to the literature, and the pectin spectra were compared to analyze structural changes. The proton peaks at 4.83–5.27 ppm and 4.24–4.75 ppm correspond to α-anomeric and β-anomeric protons, respectively [47,48]. The presence of peaks along these regions in all samples indicate that the main glycosidic linkages of pectins were not significantly altered by enzymatic treatment. The H-1 peaks of the main constituent sugars in pectin observed at 5.27, 5.12, 4.99, and 4.48 ppm were assigned to Rha, Ara, GalA, and Gal residues, respectively. The signal at 4.87 ppm corresponds to the H-1 peak of the esterified GalA residue [40]. In addition, the intense proton peaks observed around 3.83, 2.13, and 1.35 ppm were resonated from the methyl groups (–CH_3_) of esterified GalA, acetylated GalA, and Rha residues, respectively [7,19]. In PG+GL+AR-treated samples, the intensity of the peak at 3.83 ppm was notably lower (corresponding to the lower DM) than in other samples (Table 2). The two proton peaks of O-acetyl groups at 2.13 and 1.94 ppm indicate that acetyl groups were attached to *O*-2 and *O*-3 positions of GalA residues [35], respectively, and further confirm high acetylation in pectins (Table 2). Moreover, the two methyl proton peaks detected at 1.35 ppm and 1.27 ppm indicate the presence of branched and unbranched Rha residues in all pectins [11]. Overall, all pectins exhibited typical proton peaks for OKPs, as reported in previous studies [11,13,19], indicating that enzyme treatment had a negligible impact on the primary structure.

### 3.4. Molecular and Rheological Properties

The molecular weights and molecular distributions of the pectins are shown in Table 2. Untreated pectin (CON) had the highest average molecular weight (Mw) with 1868.90 kDa, while enzyme-treated pectins showed a decrease in Mw (1045.39–1276.83 kDa), according to enzyme treatments. The reduction in Mw because of hydrolysis was more observable in pectins with degraded neutral side-chains than in PG-treated pectin [49]. All pectins showed a wide molecular weight distribution with a polydispersity index Mw/Mn > 1.10 [30], and a broader distribution was observed in enzyme-treated pectins than in CON, because of the cleavage of lower molar masses. A high molecular weight average and broad molecular distribution pattern of OKPs has been reported, which are consistent with the findings of this study [10,19,20]. The molecular weight of pectins has been reported to positively influence their ES by contributing to the strong and elastic interfacial layer of the continuous phase, thereby protecting against emulsion destabilization mechanisms [8,50]. Thus, pectins with lower molecular weights have reduced surface properties and exhibit poor steric stabilization effects at the O/W interface [43].

**Figure 2:**
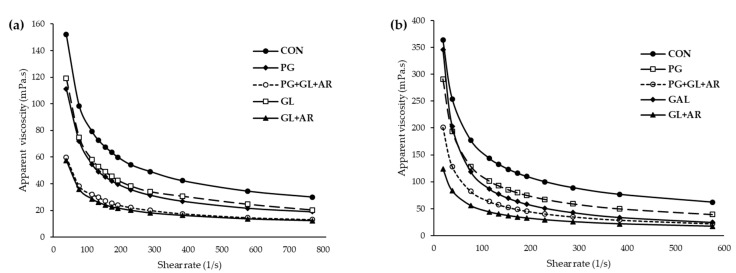
Apparent viscosity of OKP solutions (**a**) following enzymatic treatments and their corresponding emulsions and (**b**) at different shear rates.

The apparent viscosities (η_ap_) at different shear rates (γ) of the pectin solutions and their corresponding emulsions are shown in Figure 2. All pectin solutions (Figure 2a) and emulsions (Figure 2b) had similar flow patterns (decreasing η_ap_ at higher γ). The rheological parameters of the pectin solutions and emulsions were fitted to a power-law model (Equation (1)) to explain flow characteristics. All pectin solutions and emulsions exhibited shear-thinning characteristics (*n* < 1), with flow behavior index (*n*) values ranging from 0.437 to 0.559 for pectin solutions and 0.313 to 0.479 for emulsions (Table S1), as previously reported for solutions and emulsions stabilized by OKP [8,14]. However, enzyme-treated pectins showed lower apparent viscosities and consistency coefficients (*K*) than untreated pectin (Table S1), which correlated with their molecular weights. In addition, the apparent viscosities of the emulsions showed similar trends to those of their corresponding pectin solutions.

Depending on the enzymatic treatment, the sizable viscosity differences observed in pectin solutions were able to influence their emulsion activity and long-term stability. Viscosity is important for emulsifying characteristics of pectin solutions [17]. Previous studies have reported that low viscosity facilitates the initial formation of small oil droplets in emulsions produced by shearing homogenization, whereas high viscosity can reduce oil-droplet mobility and enhance ES [17,51]. However, the *n* and *K* values of the emulsions showed inconsistent trends with their corresponding pectin solutions (Table S1), implying that the rheological characteristics of the emulsions were not entirely dependent on the viscosity of the aqueous pectin phase [6]. For instance, the GL emulsion had higher values for *n* and *K* but showed lower ES than the CON and PG emulsions, indicating that additional factors other than viscosity can affect the ES of OKP.

### 3.5. Emulsion Characteristics

**Figure 3:**
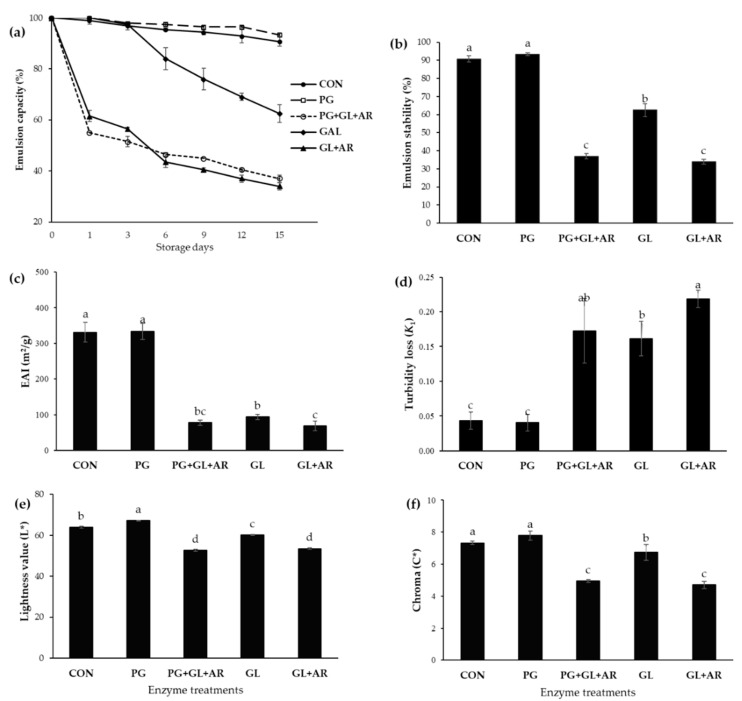
Emulsion characteristics of OKP following enzymatic treatments. Emulsion capacity during 15-day storage at 25 °C (**a**), emulsion stability (**b**), emulsion activity index EAI (**c**), and emulsion turbidity loss (**d**), after 15 days of storage at 25 °C. The lightness (**e**), and Chroma color values (**f**) of emulsions were measured immediately after preparation. Values represent mean ± standard deviation (*n* = 3), and letters (a–d) represent significant differences between samples (*p* < 0.05).

The EC at different storage intervals is shown in Figure 3a. Except for emulsions stabilized using PG-treated pectin, other enzyme-treated pectins showed lower and decreased EC throughout the storage period compared to the CON emulsions. In PG+GL+AR and GL+AR emulsions, rapid phase separation, which indicates destabilization, occurred within the first 24 h (~45% stability loss). In GL emulsions, apparent destabilization was observed after 72 h of storage at 25 °C. In contrast, the PG emulsion showed more resistance to creaming, and comparable stability to the CON emulsion during storage. At the end of the storage period, the emulsion stabilities (ES) of CON, PG, PG+GL+AR, GL, and GL+AR emulsions were 90.74%, 93.33%, 37.00%, 62.50%, and 34.00%, respectively (Figure 2b).

The EAI expresses the fraction of oil enveloped by a continuous phase (pectin solution). Thus, a high EAI value indicates the presence of more oil volume absorbed at the interface, and vice versa [35]. After storage, the oil volume at the emulsion interface was significantly different (*p* < 0.05) between the enzymatic treatments (Figure 3c). The EAI values of CON (330.99 m^2^/g) and PG (333.65 m^2^/g) emulsions were similar, while GL, PG+GL+AR, and GL+AR emulsions had significantly lower EAI values (68.45–93.87 m^2^/g). The emulsion turbidity loss (*k*_1_) indicates instability of the emulsion (emulsion breakage) during storage [52]. The high *k*_1_ values for the GL+AR (0.22), PG+GL+AR (0.16), and GL (0.14) emulsions indicated high turbidity loss and suggested poor storage stability, whereas the PG and CON emulsions showed remarkably low turbidity loss (~0.04) after the storage period (Figure 3d).

**Figure 4:**
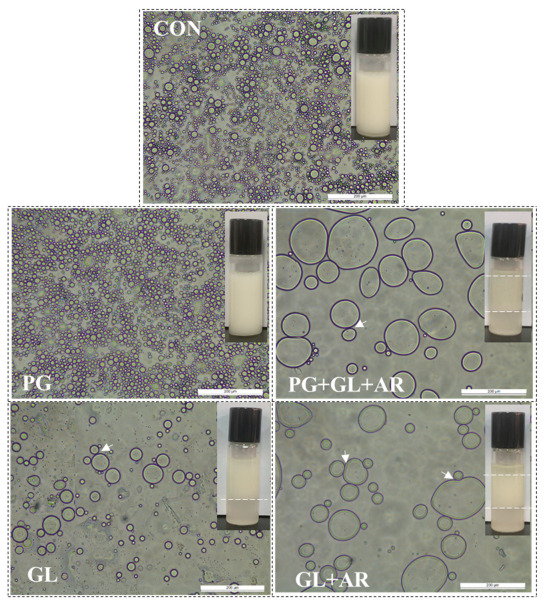
Micrographs and photographs (insets) of emulsions stabilized (pH 7.0) with enzyme-treated OKPs (0.5% *w*/*v*) after 15 days of storage at 25 °C. Oil fraction φ = 0.5. Scale bars, 200 µm. White arrows indicates droplets aggregation.

Color parameters including lightness (L* value) and chroma (C* value) have been previously used to assess emulsion characteristics [36]. The droplet characteristics (size and aggregation) are related to the lightness and chromaticity of emulsions. An increase in droplet size and aggregation results in decreasing lightness, and color intensity tends to fade [36]. In this study, we observed significant differences (*p* < 0.05) in the L* (Figure 3e) and C* values (Figure 3f) of the freshly prepared emulsions. The PG emulsion showed the highest L* and C* values, followed by the CON emulsion, and these were significantly different from those of the other emulsions (*p* < 0.05), as shown by emulsion micrographs (Figure 4).

We visualized the destabilization mechanism using microscopy (Figure 4). After storage, visible differences in droplet size and homogeneity of CON and PG emulsions compared to those of other emulsions were related to poor ES, which agrees with the results shown in Figure 3. The PG emulsions showed relatively small, uniform, and well-dispersed droplets, which explain their long-term stability. In contrast, large, non-uniformly sized, and aggregated droplets were observed in the GL, PG+GL+AR, and GL+AR emulsions resulting from flocculation and coalescence, which explain their poor ES.

### 3.6. Principal Component Analysis of Structure–Function Relationships

The relationship between the molecular structure and emulsifying properties of various pectins has been studied previously [1,3,5,24]. However, some inconsistencies exist in previous reports [1]. For instance, the presence of hydrophobic moieties, including protein, acetyl, methyl, and phenolics, in pectin structures has been linked to emulsifying properties [40,41,53], preventing aggregation or instability by forming an anchor around the surface of the oil droplets [16,27]. However, these factors do not play a prominent role and are not solely responsible for the long-term stability of OKP-stabilized emulsions [2,14].

**Figure 5:**
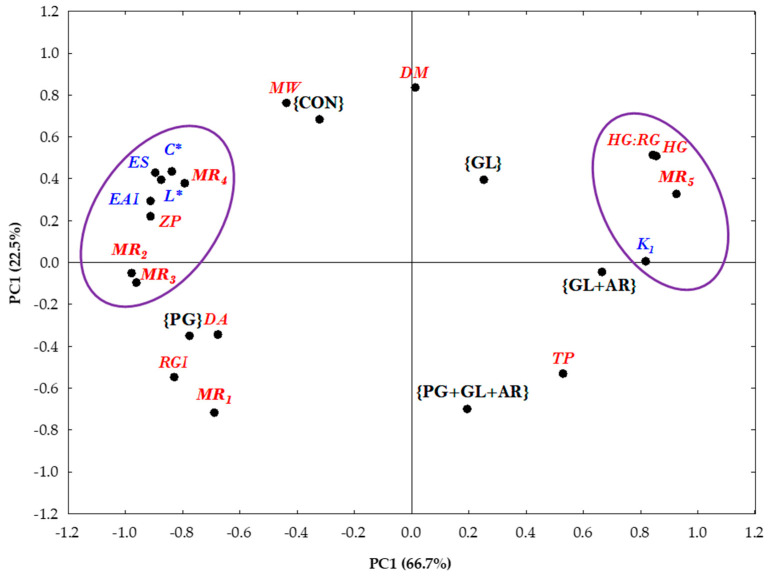
Principal component analysis (PCA) plot of OKP following enzymatic treatments (black-colored components). Red-colored and italicized components are emulsifying parameters, and blue-colored components are molar ratios. Variables within the purple circles are closely related.

In this study, the correlations between the structural parameters and emulsion characteristics were analyzed using PCA. PCA is a multivariate statistical method that performs an orthogonal transformation of multidimensional datasets into graphical visualizations consisting of components (PC1 and PC2), making the results easier to interpret. PC1 (66.7%) and PC2 (22.5%) accounted for 89.2% of the total variation, as shown in Figure 5. Pectins treated with different enzymes were grouped into separate plot areas based on distinct similarities and differences in their structural characteristics. For instance, CON- and PG-treated pectins appeared on the left axis of the PCA plot because of their comparably better emulsion characteristics. The cluster observed on the left axis of PC1 indicates that the molecular structures of MR_2_, MR_3_, and MR_4_ played significant roles in explaining the variations in the charge density (zeta potential) and emulsifying properties of OKP. Thus, the neutral side-chain sugars contributed decisively to the emulsifying properties of OKP. Moreover, the HG:RG, HG, and MR_5_ molar ratios appearing on the right axis of PC1 correlated with emulsion turbidity loss (*k_1_*), indicating a possible influence of these factors on emulsion breakage, which may explain the poor emulsifying stability of emulsions stabilized with GL-, GL+AR-, and PG+GL+AR-treated pectins appearing on the same axis.

A high proportion of RG-I in pectic polysaccharides and side-chain branching configuration contribute to long-term stability via steric stabilization [20,54,55]. However, the complete degradation of the HG domain (solely RG-I pectin) could result in deteriorated emulsifying properties [26], indicating the plausible contributions of both pectin subdomains to emulsification performance.

The high storage stability of emulsions stabilized by OKP has been attributed to their highly branched RG-I domain [22]. In addition, Kpodo et al. [2] identified the molar ratio of (Ara + Gal)/Rha as a suitable predictor of the optimum emulsification capacity of OKP. In other studies, poor emulsion stabilities wereobserved in pectins with no or low galactan and arabinan side-chain contents [43,56]. Funami et al. [27] also linked the contribution of neutral side chains in stabilizing emulsions to their ability to form a well-hydrated layer, thus facilitating long-term stability in emulsions. In addition, neutral sugar chains effectively promote steric repulsion between oil droplets, thereby preventing instability resulting from droplet aggregation and flocculation [43]. Overall, PCA revealed neutral side-chains, galactose, and arabinose as distinct molecular structures responsible for the differences in the emulsification performance of OKP, and their compositions in the pectin structure play predominant roles in the ability of OKP to form and stabilize O/W emulsions. Furthermore, no clear relationship was observed between emulsifying properties of pectins and their protein content or DM (Figure 5), which agrees with previous reports that they may not play a prominent role in the long-term stability of OKP-stabilized emulsions [2,14].

## 4. Conclusions

The influence of molecular structure on the emulsifying performance of OKPs was investigated by targeted enzymatic hydrolysis of the side chains and backbone, and important relationships were revealed using multivariate analysis. According to the enzyme treatment types, the results reflect notable differences in the backbone and side-chain structures of pectin, charge densities, acetyl and methyl contents, and significant differences in molecular masses and rheological properties. However, negligible differences were observed in protein content.

In detail, we demonstrated that the molecular structures of pectin, such as molecular weight and viscosity, might have some influence on the emulsifying properties of OKP. However, neutral side-chains of RG-I are superior contributors to the ES properties of OKP, accounting for approximately 38% (GL) and 66% (GL+AR) loss in ES after 15 days of storage at 25 °C. In addition, flocculation and coalescence were observed as the main destabilization mechanisms. On the other hand, PG treatment showed improved emulsifying properties, with smaller and well-dispersed oil droplets owing to the increase in the proportion of neutral side-chains in the modified pectin structure.

PCA analysis further confirmed that neutral sugar side chains (arabinogalactan, galactan, and arabinan) are key for long-term preservance of emulsions stabilized with OKP. Overall, our data suggest that the emulsifying properties of OKP rely on its molecular composition and structure. It is thus proposed that an extraction protocol tailored to preserving or enriching the proportion of neutral sugar side-chain structures is beneficial in obtaining OKP with good emulsification performance, which can be used as a natural emulsifier in the food industry.

## Figures and Tables

**Table 1:** Enzyme treatment description.

Treatment Code	Treatment Description
CON	Okra pectin (OKP) obtained by hot sodium acetate buffer (0.05 M, pH 4.5) extraction.
PG	OKP hydrolyzed with endo-polygalacturonanase (0.4 U/mg) enzyme
PG+GL+AR	OKP hydrolyzed with endo-polygalacturonanase (0.4 U/mg), endo-1,4-β-galactanase (0.4 U/mg), β-galactosidase (0.4 U/mg), endo-1,5-α-arabinanase (0.2 U/mg), and α-l-Arabinofuranosidase (0.2 U/mg) enzymes
GL	OKP hydrolyzed with endo-1,4-β-galactanase (0.4 U/mg) and β-galactosidase (0.4 U/mg) enzymes
GL+AR	OKP hydrolyzed with endo-1,4-β-galactanase (0.4 U/mg), β-galactosidase (0.4 U/mg), endo-1,5-α-arabinanase (0.2 U/mg), and α-l-arabinofuranosidase (0.2 U/mg) enzymes.

**Table 2:** Physicochemical properties and monosaccharide composition of OKPs following enzymatic hydrolysis ^1,2^.

	CON	PG	PG+GL+AR	GL	GL+AR
Recovery ^1^ (%)	-	80.26 ± 0.90 ^b^	71.20 ± 0.36 ^d^	82.36 ± 0.54 ^a^	78.09 ± 1.26 ^c^
Total protein (%)	1.56 ± 0.04 ^b^	1.53 ± 0.04 ^b^	1.66 ± 0.04 ^a^	1.57 ± 0.02 ^b^	1.60 ± 0.05 ^ab^
Degree of acetylation ^2^ (%)	43.23 ± 0.66 ^c^	47.45 ± 0.07 ^a^	46.61 ± 0.54 ^a^	44.83 ± 0.23 ^b^	37.70 ± 0.28 ^d^
Degree of methylation ^2^ (%)	14.59 ± 1.11 ^a^	8.13 ± 0.37 ^d^	6.05 ± 0.13 ^c^	10.96 ± 0.05 ^b^	7.89 ± 0.57 ^c^
Zeta potential (mV)	−34.40 ± 1.86 ^bc^	−39.60 ± 1.56 ^c^	−25.54± 1.81 ^a^	−31.42 ± 2.69 ^b^	−21.15 ± 2.33 ^a^
Monosaccharides ^3^ (g/100 g)					
Mannose	0.32 ± 0.07 ^a^ (0.4)	-	-	-	-
Rhamnose	13.44 ± 0.09 ^a^ (15.0)	13.68 ± 0.26 ^a^ (18.4)	12.35 ± 0.58 ^b^ (18.4)	10.03 ± 0.53 ^d^ (16.0)	11.26 ± 0.03 ^bc^ (16.2)
Galacturonic acid	47.91 ± 1.08 ^a^ (53.6)	35.85 ± 0.09 ^c^ (48.2)	36.49 ± 0.27 ^c^ (54.3)	37.03 ± 0.32 ^c^ (59.0)	41.88 ± 2.12 ^b^ (60.2)
Glucose	0.88 ± 0.01 ^a^ (1.0)	0.69 ± 0.07 ^b^ (0.9)	0.44 ± 0.02 ^c^ (0.7)	0.34 ± 0.05 ^d^ (0.5)	0.47 ± 0.02 ^c^ (0.7)
Galactose	25.66 ± 0.28 ^a^ (28.7)	22.93 ± 0.23 ^b^ (30.8)	17.49 ± 0.18 ^c^ (26.0)	14.40 ± 0.25 ^e^ (23.0)	15.63 ± 0.30 ^d^ (22.5)
Arabinose	1.17 ± 0.03 ^b^ (1.3)	1.22 ± 0.02 ^a^ (1.7)	0.40 ± 0.03 ^c^ (0.6)	0.97 ± 0.04 ^b^ (1.6)	0.37 ± 0.00 ^c^ (0.5)
Molecular parameters					
Number Average Mn (kDa)	471.02 ± 12.13 ^a^	178.17 ± 0.82 ^d^	210.48 ± 7.57 ^c^	215.61 ± 1.12 ^b^	156.57 ± 2.27 ^e^
Number Average Mw (kDa)	1868.90 ± 18.80 ^a^	1209.39 ± 25.22 ^c^	1091.54 ± 3.18 ^d^	1276.83 ± 17.78 ^b^	1045.39 ± 7.56 ^e^
Polydispersity (Mw/Mn)	3.97	6.79	5.19	5.92	6.68

Values show the mean ± standard deviation (*n* = 3), and letters (a–e) represent significant differences between samples (*p* < 0.05). ^1^ The recovery was calculated as the % ratio of initial pectin weight before and after enzyme treatment. ^2^ mol of acetic acid and methanol per 100 mol of total uronic acid. ^3^ Values in parentheses are molar percentage (mol%).

**Table 3:** Molar ratios of OKPs following enzymatic treatments.

	Molar Ratios ^1^							
	MR_1_	MR_2_	MR_3_	MR_4_	MR_5_	HG	RG-I	HG/RG-I
CON	0.28	0.44	0.42	0.02	1.19	38.56	60.10	0.64
PG	0.38	0.49	0.46	0.02	0.95	29.80	69.27	0.43
PG+GL+AR	0.34	0.37	0.36	0.01	1.21	35.93	63.41	0.57
GL	0.27	0.33	0.31	0.02	1.46	43.02	56.44	0.76
GL+AR	0.27	0.30	0.29	0.01	1.54	43.96	55.37	0.80

^1^ Molar ratios were measured as follows: Contribution of RG-I region to pectin backbone structure MR_1_ = Rha/GalA; ratio of side-chains in backbone MR_2_ = (Gal + Ara)/(Rha + GalA); ratio of Gal in backbone MR_3_ = Gal/(Rha + GalA); ratio of Ara in backbone MR_4_ = Ara/(Rha + GalA); linearity of backbone MR_5_ = GalA/(Rha + Gal + Ara); proportion of HG in backbone = GalA – Rha; and proportion of RG-I = 2 × Rha + Ara + Gal.

## Data Availability

All related data and methods are presented in this paper. Additional inquiries should be addressed to the corresponding author.

## Supplementary Materials

The following supporting information can be downloaded at https://www.mdpi.com/article/10.3390/foods11101497/s1, Table S1: flow behavior index (*n*), consistency index (κ), and apparent viscosity (η_ap_) of pectin solutions and their corresponding oil-in-water emulsions.
