# Supplementary material for: Enzymatic Hydrolysis Modifies Emulsifying Properties of Okra Pectin
Source: Foods. 2022 May 20;11(10):1497. doi: 10.3390/foods11101497 (PMC9140940; doi:10.3390/foods11101497)
Supplement: Supplementary file 1 [file foods-11-01497-s001.zip › foods-1706878-supplementary.pdf]

Supplementary file: Enzymatic hydrolysis modifies emulsifying properties of okra pectin

Table S1. Flow behavior index (n), consistency index (κ), and apparent viscosity (η_{ap}) of pectin solutions and their corresponding oil-in-water emulsions.

Model parameters	Pectin solutions				Emulsions			
	K	*n*	r^2	[1]η_{ap}	*K*	*n*	r^2	[1]η_{ap}
**CON**	8.928	0.488	0.998	59.85 ± 0.16[a]	16.976	0.479	0.995	111.30 ± 0.32[a]
**PG**	7.656	0.440	0.995	38.58 ± 0.23[c]	16.573	0.411	0.996	75.35 ± 0.26[b]
**PG+GL+AR**	2.843	0.535	0.996	24.08 ± 0.26[d]	14.047	0.347	0.993	44.63 ± 0.59[d]
**GL**	8.249	0.437	0.994	42.50 ± 0.71[b]	18.191	0.318	0.990	55.88 ± 0.25[c]
**GL+AR**	2.263	0.559	0.995	21.70 ± 0.22[e]	6.902	0.420	0.995	31.90 ± 0.65[e]

[1]η_{ap} recorded at a shear rate of 192 s^{-1} (50 rpm). Data presented as mean ± standard deviation (n = 3), and letters (a-e) represent significant differences between samples ($p < 0.05$).
